# Development and ELISA Characterization of Antibodies against the Colistin, Vancomycin, Daptomycin, and Meropenem: A Therapeutic Drug Monitoring Approach

**DOI:** 10.3390/antibiotics13070600

**Published:** 2024-06-27

**Authors:** Vivian Garzon, J.-Pablo Salvador, M.-Pilar Marco, Daniel G.-Pinacho, Rosa-Helena Bustos

**Affiliations:** 1 Doctoral Programme of Biosciences, Universidad de La Sabana, Chía 140013, Colombia; viviangaru@unisabana.edu.co; 2 Therapeutic Evidence Group, Clinical Pharmacology, Universidad de La Sabana, Chía 140013, Colombia; daniel.g.pinacho@gmail.com; 3 CIBER de Bioingeniería, Biomateriales y Nanomedicina (CIBER-BBN), 08034 Barcelona, Spain; jpsqob@cid.csic.es (J.-P.S.); pilar.marco@cid.csic.es (M.-P.M.); 4 Nanobiotechnology for Diagnostics (Nb4D), Department of Surfactants and Nanobiotechnology, Institute for Advanced Chemistry of Catalonia (IQAC) of the Spanish Council for Scientific Research (CSIC), 08034 Barcelona, Spain; 5 Clínica Universidad de La Sabana, Chía 140013, Colombia

**Keywords:** antibiotics, antimicrobial resistance (AMR), Daptomycin, Vancomycin, Meropenem, Colistin, antibodies, ELISA

## Abstract

More than 70% of bacteria are resistant to all or nearly all known antimicrobials, creating the need for the development of new types of antimicrobials or the use of “last-line” antimicrobial therapies for the treatment of multi-resistant bacteria. These antibiotics include Glycopeptide (Vancomycin), Polymyxin (Colistin), Lipopeptide (Daptomycin), and Carbapenem (Meropenem). However, due to the toxicity of these types of molecules, it is necessary to develop new rapid methodologies to be used in Therapeutic Drug Monitoring (TDM). TDM could improve patient outcomes and reduce healthcare costs by enabling a favorable clinical outcome. In this way, personalized antibiotic therapy emerges as a viable option, offering optimal dosing for each patient according to pharmacokinetic (PK) and pharmacodynamic (PD) parameters. Various techniques are used for this monitoring, including high-performance liquid chromatography (HPLC), gas chromatography-mass spectrometry (GC-MS), and immunoassays. The objective of this study is the development and characterization by ELISA of specific polyclonal antibodies for the recognition of the antibiotics Vancomycin (glycopeptide), Colistin (polymyxin), Daptomycin (lipopeptide), and Meropenem (carbapenem) for future applications in the monitoring of these antibiotics in different fluids, such as human plasma. The developed antibodies are capable of recognizing the antibiotic molecules with good detectability, showing an IC50 of 0.05 nM for Vancomycin, 7.56 nM for Colistin, 183.6 nM for Meropenem, and 13.82 nM for Daptomycin. These antibodies offer a promising tool for the precise and effective therapeutic monitoring of these critical antibiotics, potentially enhancing treatment efficacy and patient safety.

## 1. Introduction

Antimicrobial resistance (AMR) has become one of the main public health problems in recent years, due to the inappropriate and excessive use of antibiotic agents both in the clinical and veterinary fields, that have led to an increase in gene mutations bacteria and their horizontal transfer [1,2]. According to the World Health Organization (WHO), it is estimated that in 2050, infections caused by bacteria resistant to antibiotics drugs could cause the death of 10 million people worldwide each year at a cost of around USD 100 trillion in losses for production of new molecules [3]. This has led to the need to implement a series of measures to minimize the impact [4], such as prescribing and dispensing antibiotics only when necessary or avoiding infections by training and ensuring the cleanliness of hands, instruments, and the environment [5,6].

However, the measures to reduce the increase in AMR infections have not been sufficient [2]; on the contrary, it has been increasing, affecting not only the health of patients but also the financial stability of health systems worldwide [7]. This increase in AMR bacteria has required the development of new types of antibiotics or the use of antibiotic therapies with highly toxic “last-line” drugs such as Colistin [8,9].

Due to the above, therapeutic drug monitoring (TDM) became necessary, a tool of clinical importance defined as the individualization of the dose of a drug [10], maintaining the drug concentrations in body fluids (blood, plasma, urine, among others) within from a target range [11]. This monitoring is carried out using different techniques, including high-performance liquid chromatography (HPLC-MS/MS) [12], (HPLC-UV) [13], gas chromatography-mass spectrometry (GC-MS) [14,15], immunoassays [16,17], and biosensors [8,18]. Immunoassays are one of the most widely used and are based on antigen–antibody binding (immunocomplex) for the detection of analytes in different samples [19]. The main immunoassays used in TDM are radioimmunoassays (RIAs), fluoroimmunoassays (FPIAs) and enzyme-linked immunosorbent assays (ELISAs), which have been allowed the quantification of antibiotics, anticancers, and biologic drugs [20,21,22].

The ELISA technique has been widely used for the evaluation of different molecules in different matrices, using monoclonal or polyclonal antibodies developed specifically for each molecule. However, at the clinical level, there is not a wide commercial availability of antibodies against antibiotics [23,24,25]. ELISA assays have been used for the detection of antibiotic molecules in different matrices such as quinolones in milk [26], marine products and eggs [27], penicillin and chloramphenicol in honey [28], polymyxin B in human serum [29], ceftazimide in urine [30], Vancomycin in blood [16], Colistin in residue porcine and poultry muscle [31], Daptomycin in human serum [32], among others. A careful design and synthesis of the immunizing hapten is necessary for the development of polyclonal or monoclonal antibodies in animal models, preserving as much as possible most of the steric and electronic properties of the analyte [19,33]. The antibodies also could be used in immunosensors detection of aminoglycosides in blood [34], tobramycin in serum [35], tetracycline in urine [36], and Vancomycin in plasma [37].

**Figure 1:**
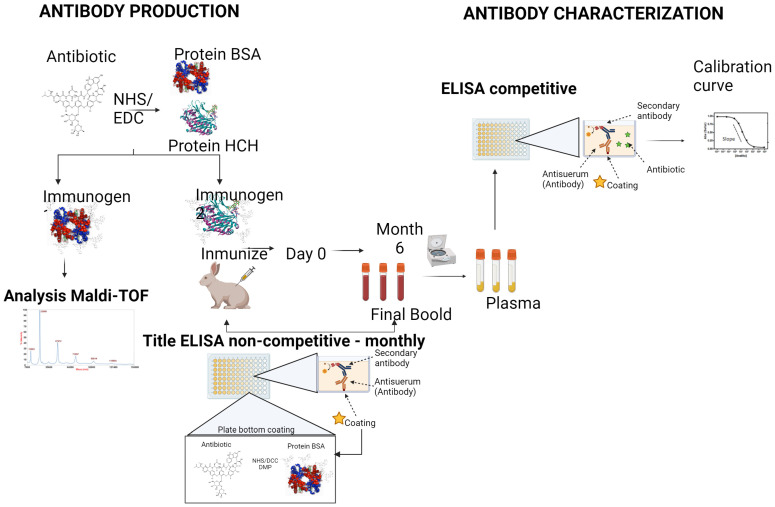
Graphical abstract. Conceptual design overview of the production of antibodies.

The aim of this work is the development and characterization by ELISA of specific antibodies for the recognition of Vancomycin (glycopeptide), Colistin (polymyxin), Daptomycin (lipopeptide), and Meropenem (carbapenem) antibiotics for future applications for monitoring these antibiotics in different fluids, such as human plasma or urine, through the use of immunoassay or immunosensors (See Figure 1).

## 2. Materials and Methods

### 2.1. Reagents and Instruments

#### General Methods and Instruments

The MALDI-TOF-MS (matrix-assisted laser desorption ionization time-of-fly mass spectrometer) used for analyzing the protein conjugates was a Bruker Microflex LT Bruker Daltonics, Bremen, Germany) provided with the software Voyager-DE-RP (version 4.03) developed by Perspective Biosystems Inc. (Framingham, MA, USA). The pH and the conductivity of all buffers and solutions were measured with a pH meter pH 540 GLP and a conductometer LF 340, respectively (WTW, Weilheim, Germany).

Polystyrene microtiter plates were purchased from Nunc (Maxisorp, Roskilde, Denmark). Washing steps were performed on an SLY96 PW microplate washer (SLT Labinstruments GmbH, Salzburg, Austria). Absorbances were read on a Thermo Scientific MultiSkan GO (Thermo Fisher Scientific, Waltham, MA, USA) at a single wavelength mode (450 nm). The competitive curves were analyzed with a four parameter logistic equation using the software GraphPad Prism 7.0 (GraphPad Software Inc., San Diego, CA, USA) according to the following formula: y = B(A − B)/[1 − (x/C)D], where A is the maximum absorbance, B is the minimum absorbance, C is the concentration producing 50% of the maximal absorbance, and D is the slope at the inflection point of the sigmoid curve. Unless otherwise indicated, the data presented correspond to the average of at least two well replicates.

### 2.2. Chemicals and Immunochemicals

Colistin (COL) and Vancomycin (VAN) were supplied by Sigma-Aldrich (St. Louis, MO, USA). Daptomycin (DAP) (Cubicin^®^ 350 mg) was supplied by Novartis and Meropenem (MER) was obtained by Farmacologíca S.A. Bovine serum albumin (BSA), horseshoe crab hemocyanin (HCH), Freund’s complete and incomplete adjuvants (cFA and iFA), 1-ethyl-3-(dimethylaminopropyl)carbodiimide hydrochloride (EDC), N-hydroxysuccinimide (NHS), dicyclohexyl carbodiimide (DCC), and Dimethyl pimelimidate (DMP) were supplied by Sigma-Aldrich (St. Louis, MO, USA). HiTrap™ Desalting was supplied by GE Healthcare Bio-Sciences AB, (Uppsala, Sweden). Dimethylformamide (DMF) and Trifluoroacetic acid (TFA) were supplied by Sigma-Aldrich (St. Louis, MO, USA).

Buffers: Phosphate-buffered saline (PBS) is 0.01 M phosphate buffer on a 0.8% saline solution, and the pH is 7.5. PBST is PBS with 0.05% Tween 20. Borate buffer is 0.2 M boric acid/sodium borate, pH = 8.7. Coating buffer is 0.05 M carbonate-bicarbonate buffer, pH 9.6. Citrate buffer is a 0.04 M solution of sodium citrate, pH 5.5. The substrate solution contains 0.01% TMB (3,3′,5,5′- tetramethylbenzidine) and 0.004% H_2_O_2_ in citrate buffer.

### 2.3. Antibody Development Preparation of Bioconjugates

#### 2.3.1. Immunogens

The haptens COL, VAN, DAP, and MER were coupled to the proteins (BSA and HCH) by the EDC/NHS method previously described [26,38,39]. Briefly, proteins (10 mg) were dissolved in 800 µL of 1X PBS and a solution of hapten (10 μmol) in PBS was added. Then, NHS solution (18 µmol) in 1X PBS was added, followed by the dropwise addition of EDC solution (18 µmol) in PBS. The pH of final solutions was adjusted to 9 and the mixtures were stirred overnight at room temperature (RT) (Figure 1).

The protein conjugates were purified by size-exclusion chromatography using a HiTrap desalting Sephadex G-25 Superfine column (Amersham Biosciences) following the manufacturer’s instructions, using ultrapurewater as eluent and stored freeze-dried at −80 °C. Unless otherwise indicated, working aliquots were stored at 4 °C in PBS at 1 mg/mL.

#### 2.3.2. Coating Antigens

The production of polyclonal antibodies for COL was carried out in the vivarium of the Pontifical Javeriana University (Bogotá) and the production of antibodies for VAN, DAP, and MER has been carried out with the support of U2 (Custom Antibody Service (CAbS) of the ICTS “NANOBIOSIS” (Spain). The immunization protocol was performed on female New Zealand white rabbits weighing 1–2 kg. Animals were immunized using the horseshoe crab hemocyanin (HCH) bioconjugate. Approval was given by the bioethics committee of the Pontificia Universidad Javeriana, Bogota, according to the guidelines given in FUA 072-18 (13 February 2019).

Briefly, 12 rabbits were immunized using COL-HCH, Rb66-Rb67-Rb72; VAN-HCH; Rb157—Rb158—Rb159; DAP-HCH, Rb154—Rb155—Rb156, and MER-HCH Rb160—Rb161—Rb162. The immunization schedule was for 6 months according to the following procedure: (i) 100 μg of immunogen per rabbit in complete Freund’s adjuvant (1:1) intradermally on day 0; (ii) the same concentration of immunogen in incomplete Freund’s adjuvant (1:1) intradermally once a month for 6 months. The rabbits were bled through the marginal veins of the ear 10 days after each of the inoculations of procedure (ii) to monitor the process and the final bleeding was performed after 6 months by cardiac puncture. Serum was obtained by centrifugation at 4500 rpm for 5 min [19,40].

#### 2.3.3. Preparation of Coating Antigens

##### DCC/NHS

The haptens possessing carboxylic acid group (VAN, DAP, and MER) were coupled to BSA protein by the ester active method previously described [19,39,41]. VAN, DAP, and MER were dissolved in DMF and mixed with 50 µmol NHS and 100 µmol DCC both dissolved in DMF. The mixtures were allowed to react under magnetic stirring for 3 h at RT, until the solutions became muddy due to the urea precipitation. Then, solutions were centrifuged (10,000 rpm for 10 min) to remove the precipitate and supernatants were added to solutions of the protein (10 mg) in borax/borate buffer (100 µL). The reaction mixtures were kept at RT with magnetic stirring overnight. Finally, the mixtures were purified by dialysis against 0.05 mM PBS (4 times × 5 L) and Milli-Q water (1 time × 5 L) and stored lyophilized at −80 °C. Working aliquots were stored at 4 °C in 0.01 M PBS at 1 mg/mL.

##### Dimethyl Pimelimidate (DMP)

Haptens COL and DAP were also coupled to BSA protein using homobifunctional imidoesters [42,43]. Two solutions were prepared separately: (A) dimethylpimellimidate hydrochloride (DMP 2 HCl, 0.16 mg) was dissolved in borate buffer 0.2 M and (B) COL or DAP (0.33 mg) were dissolved in ultrapurewater both in ice bath. Then, these solutions were mixed and the final mixture was immediately added dropwise to a BSA solution (2 mg) in borax/borate buffer 0.2 M, kept stirring for 10 min in ice bath and 60 min at RT. The reaction was stopped by adding Tris base (20 mM) in borate buffer 0.2 M. Finally, the conjugates were purified by dialysis against 0.5 mM PBS (4 times × 5 L) and ultrapurewater (1 times × 5 L) and stored lyophilized at −80 °C. Working aliquots were stored at 4 °C in 0.01 M PBS at 1 mg/mL [44]. Table S1 summarizes the different bioconjugates obtained according to hapten and protein combination and bioconjugation method used.

##### Hapten Density Analysis

Hapten densities of BSA protein conjugates were calculated by MALDI-TOF MS by comparing the molecular weight of the native proteins with the bioconjugates. Spectra were obtained by mixing 2 μL of the freshly prepared matrix (sinapinic acid, 10 mg mL^−1^ in ACN, 0.1% TFA) with 2 μL of a solution of the BSA conjugates in ACN/H_2_O 50:50, 0.1% TFA (5 mg/mL) (See Table 1). The hapten density (δ hapten) was calculated according to the following equation:

[MW(Conjugate) − MW(Protein)]/MW(Hapten).

### 2.4. Polyclonal Antibodies

The characterization of the polyclonal antibodies (pAbs) obtained was performed by indirect ELISA.

#### 2.4.1. Antibody Titer

Different measurements of the titers over time of the partial bloods were carried out. Microtiter plates were coated with the corresponding BSA-EDC coating antigen in coating buffer (10 μg/mL, 100 µL/well) overnight at 4 °C and covered with adhesive plate sealers. The next day, the plates were washed with PBST (four times, 300 μL/well) followed by the addition of the corresponding antisera in PBST with a dilution range of 1/1000 to 1/64,000 (100 μL/well) to the microtiter plates. After a 30 min incubation time at RT, the plates were washed again as before and a solution of anti IgG-HRP (1/6000 in PBST) was added (100 µL/well) and incubated for a further 30 min. at RT. The plates were washed again and the substrate solution (100 µL/well) was added. Color development was stopped after 30 min at RT with 4 N H_2_SO_4_ (50 µL/well) and absorbances were read at 450 nm.

#### 2.4.2. Two-Dimensional Titration Assays

Two-dimensional (2D) titration assays were developed using all available bioconjugates by using several antigen concentration and antiserum dilutions, with the aim of obtaining the optimal concentrations of both immunoreagents to perform the competitive assays.

Microtiter plates were coated with the corresponding coating antigen in coating buffer (100 µL/well) overnight at 4 °C and covered with adhesive plate sealers. Concentrations for each bioconjugate ranged from 10 μg/mL to 0.01562 μg/mL. The following day, the plates were washed with PBST (four times, 300 μL/well) followed by the addition of the corresponding antisera in PBST (100 μL/well) in a dilution range of 1/1000 to 1/64,000 microtiter plates. After a 30 min incubation time at RT, the plates were washed again as before and a solution of anti IgG-HRP (1/6000 in PBST) was added (100 µL/well) and incubated for a further 30 min at RT. The plates were washed again and the substrate solution (100 µL/well) was added. Color development was stopped after 30 min at RT with 4 N H_2_SO_4_ (50 µL/well) and absorbances were read at 450 nm.

The concentrations correspond to those that generate absorbance values close to 1 unit at a concentration of coating antigen corresponding to 70–80% of the saturation which were selected for competitive assays (Table 2). These values have been reported in the literature, corresponding to a zone of high stability providing better detection of analyte [33].

#### 2.4.3. Competitive Assay

Microtiter plates were coated with the corresponding coating antigen in coating buffer (100 μL/well) overnight at 4 °C and covered with adhesive plate sealers. The optimal concentrations for each immunoreagent are shown in Table 2.

The following day, the plates were washed with PBST (four times, 300 μL/well) and the corresponding antibiotics VAN, COL, DAP, MER standards (0.01 nM–10,000 nM, in PBST, 50 μL/well) followed by corresponding antisera (see Table 2 for concentrations in PBST, 50 μL/well) were added to the microtiter plates. After 30 min of incubation time at RT, the plates were washed again as before and a solution of anti IgG-HRP (1/6000 in PBST) was added (100 μL/well) and incubated for 30 min more at RT. The plates were washed again, and the substrate solution was added (100 μL/well). Color development was stopped after 30 min at RT with 4 N H_2_SO_4_ (50 μL/well), and the absorbances were read at 450 nm.

## 3. Results

### 3.1. Preparation of Immunogens

To carry out the conjugation of the antibiotics to the protein, it is necessary to evaluate their chemical structure to find the best linking technique without affecting their properties and structure. The resulting conjugates are used for immunization of the host animals and the antibodies obtained are screened in immunoassays such as the Enzyme-Linked ImmunoSorbent Assay (ELISA) [24].

**Figure 2:**
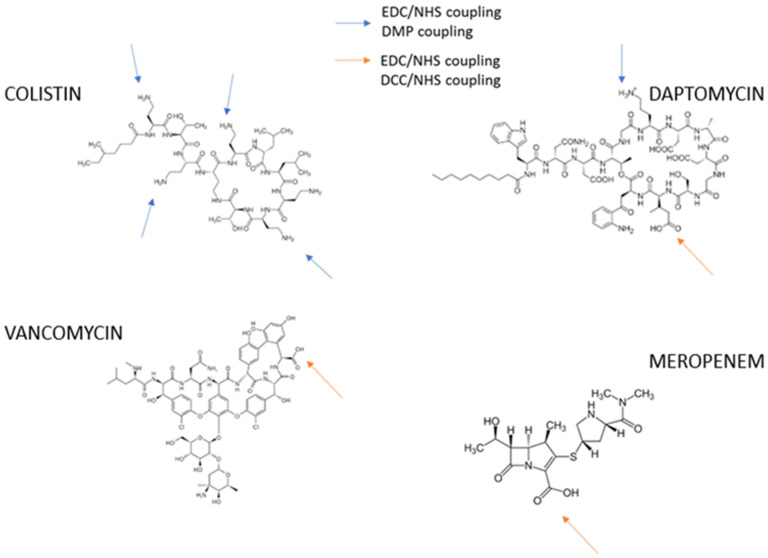
Chemical structure of immunogens (coupling the antibiotics to BSA and HCH proteins).

To induce an appropriate immune response in the host animal, immunogens were prepared using an EDC/NHS active ester method by coupling the antibiotics to BSA and HCH proteins. Figure 2 shows the structure of the antibiotics with carboxylic acid and amino groups that interact with groups of the protein.

In the case of VAN-EDC-HCH and COL-EDC-HCH, a precipitate was observed during conjugation, due to the decrease of the pH to 4. For this reason, the pH was adjusted to 8.6 using NaOH (initial pH of the Borate medium) [45,46]. As pH is a determining parameter in the bioconjugation, it was monitored before and after conjugation [47].

#### 3.1.1. Preparation of Coating Antigens: Active Ester DCC/NHS and DMP Bioconjugates

The format chosen for the development of the different ELISA proposed was an indirect format which requires the preparation of protein bioconjugates, such as BSA, called coating antigens (Figure 2). In the case of COL and DAP, the amines of the antibiotics and proteins were also crosslinked using DMP in a borate buffer [48].

#### 3.1.2. Characterization by MALDI-TOF MS

Hapten densities for each BSA bioconjugate were determined using MALDI-TOF MS, by comparison of molecular weight of each bioconjugate with that of the native protein [19]. Due to their high molecular weight of HCH, it is very difficult to ionize those HCH bioconjugates, so no results were obtained [49,50]. In order to ensure the success of the bioconjugation, a BSA conjugate was prepared in parallel and characterized these BSA conjugates. Also, EDC/NHS BSA conjugates can be used as coating antigens for the characterization of the antibodies and evaluation of the antibody titer. In the case of DAP-EDC-BSA, no peak in MALDI was observed, but once the first bleeding was obtained from DAP-EDC-HCH immunization, it revealed a positive response, demonstrating the conjugation of DAP to HCH. In all cases, bioconjugation took place with a enough hapten density to induce an immune response (Table 1).

### 3.2. Characterization of Polyclonal Antibodies against Antibiotic

#### 3.2.1. Antibody Production and Antibody Titer

**Figure 3:**
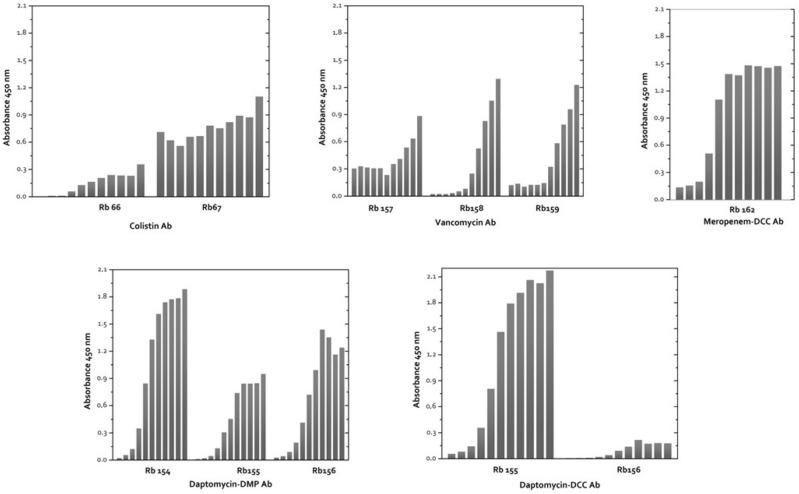
1D ELISA heterologous competitors COL-BSA-DMP, VAN-BSA-DCC, DAP-BSA-DMP, DAP-BSA-DCC, and MER-BSA-DCC. Plates were coated at 1 μg/mL of heterologous competitor bioconjugate , and antisera were diluted 1/1000 to 1/64,000.

Once the immunogens were obtained and characterized, three rabbits per immunogen were immunized using COL-EDC-HCH, VAN-EDC-HCH, DAP-EDC-HCH, and MER-EDC-HCH bioconjugates to obtain polyclonal antisera against Colistin, Vancomycin, Daptomycin, and Meropenem, respectively. Antibody titer evolution was evaluated by an indirect non-competitive ELISA making several dilutions of the antisera (dilution 1/1000 to 1/64,000) (Figure 3).

#### 3.2.2. ELISA Development

Homologous competitors (COL-EDC-BSA, VAN-EDC-BSA, DAP-EDC-BSA, and MER-EDC-BSA) were tested by carrying out 2D ELISA and competitive ELISA. However, it was decided to use the heterologous competitors (COL-DMP-BSA, VAN-DCC-BSA, DAP-DMP-BSA, DAP-DCC-BSA, and MER-DCC-BSA) against the antisera that showed a greater response in the previous test (Table S2).

The avidity of antisera (EDC) and BSA heterologous bioconjugates (HCH) were evaluated by two-dimensional titration assays from which the most suitable concentrations for each antiserum/bioconjugate competitor combination were selected. In general, the heterologous combinations obtained higher absorbances compared to the homologous assays because the heterologous ones demonstrate more affinity to the antibody as observed in Figure S2 where the absorbances are closer to the *y*-axis in two-dimensional titration assays.

#### 3.2.3. Indirect Competitive ELISA Immunoassay

**Figure 4:**
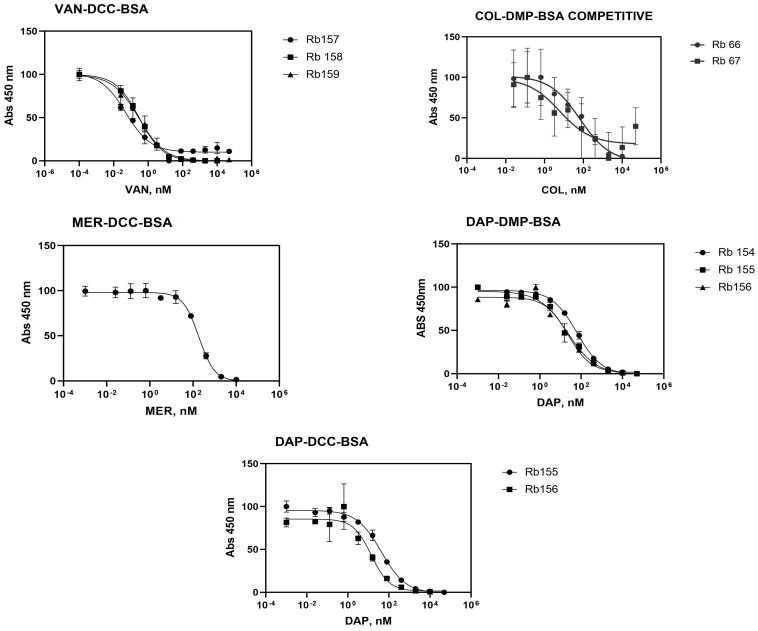
Indirect competitive ELISA immunoassay. Taking into account the concentrations obtained in the two-dimensional tests. Average calibration curve.

The optimal combinations of immunoreagents were assessed on an indirect competition assay for their ability to recognize their corresponding analyte antibiotic (Figure 4). Plates were coated using the chosen concentration of antigen and the antibody competes with several concentrations of the antibiotic in solution [51]. The results of all antisera were fitted to a four parameter equation and characteristic parameters were determined (Table 3) as the IC_50_ value, which refers to the concentration that reduces the maximum signal of the curve by 50%, giving an indirect estimation of the affinity of the antibody for the analyte [19].

The antisera Rb155 and Rb156 for Daptomycin showed an IC_50_ lower than Rb154, so these two antibodies exhibit better detectability of the analyte. The results for Rb156 are more like DAP-DMP.

## 4. Discussion

Antibiotics are low molecular weight molecules, usually less than 2000 Da, which are in general not immunogenic. Therefore, they do not induce an immune response unless they are combined with macromolecules, such as high molecular weight proteins like hemocyanins, serum albumins, thyroglobulin, fibrogen, hemocyanins or ovalbumin [52]. To induce the immune response in the animal, the NHS/EDC active ester method was carried out by coupling the antibiotics to the proteins. This method consisted in the formation of an amide bond between the carboxylic acid of the antibiotics and the amine groups of the lysines on the surface of the proteins, and between the haptens possessing amino groups and the carboxylic acids available in the surface of proteins [53]. Among the most important parameters is the pH, since a pH above 8.0 favors the formation of stable bonds of the amino groups present in antibiotics or proteins [45,46]. In the literature, conjugations with NHS/EDC have been found to obtain bioconjugates with different molecules [54,55,56].

For the coating antigens, we proposed the preparation of alternative coating antigens by a different coupling method. The coating antigens were prepared with the aim of avoiding cross-recognition of the conjugation by-products by the generated antiserum. The DCC/NHS method was chosen for DAP, VAN, and MER, which incorporate a carboxylic acid in their structure (Figure 2). The DCC/NHS method specifically activates the carboxylic acid moiety of the antibiotics, allowing chemical coupling to the free amino acids of BSA, unlike the EDC/NHS methods which are used for the couplings of amino acids and carboxylic acids of antibiotics and proteins in a one-step reaction. For the reaction of DMP being a homobifunctional agent that has both imidoester groups, these groups are reactive with amines resulting in a stable amidine.

To characterize these immunogens, MALDI-TOF was used, finding that the higher the hapten density, the greater the hapten binding to the protein and, in most cases, it will favor the production of antibodies by the animal host [57,58]. As observed in the results, MER-EDC-BSA and MER-DCC-BSA presented a higher hapten density than the other conjugates, maybe because it is more reactive towards the protein due to its lower size (See Figure S1). On the other hand, the hapten densities are very similar by comparing the same antibiotic using a different carbodiimide, so the carbodiimide type has no influence in the hapten density obtained.

The titers of the antisera were evaluated by indirect non-competitive ELISA. For the ELISA tests, the heterologous competitors were used. Since the heterologous competitors are not identical to the homologues used in rabbit immunization, they avoid the recognition of protein-bound by-products during bioconjugation, providing a higher specific recognition of the antibiotics by the antibody. Two types of conjugation were performed with DMP and DCC taking into account the functional groups (amino acids and carboxylic acids) present in the antibiotics.

For the indirect competitive ELISA immunoassay, initially, the ELISA assays were evaluated with the antigens synthesized from the NHS/EDC, the homologous ones with respect to the immunogen. The results obtained showed a very high absorbance but there was no evidence of competition by the antibodies of any of the antibiotics. A possible reason would be that some clones of the antisera developed showed a strong affinity for the by-products of conjugation attached to the protein, generating a high noise signal that hides the competition of clones recognizing the antibiotic [59,60,61].

Due to the above, heterologous antigens were tested to avoid the recognition of the by-products of bioconjugation. In this case, the competition of antibodies was observed for all antibiotics (Figure 4). COL-DMP-BSA has the highest errors in the competitive ELISA assays. We believe that this may be due to the lower affinity of this antibody for the analyte, as indicated by the shallow slope of the curve, which results in varying values in the replicates. However, this antibody is capable of detecting the Colistin analyte at concentrations appropriate for therapeutic levels in plasma samples from patients. For VAN-DCC-BSA, a lower IC50 was obtained for Rb 157, possibly due to the higher noise level than for Rb158 and Rb159. According to the above, it can be deduced that the Rb 157 antiserum has a high detectability of Vancomycin. According to the literature, Vancomycin quantification studies using monoclonal antibodies have been reported, mainly in foods such as milk enriched with antibiotics with an IC50 of 0.00948 nM; this is lower than that obtained in our assay, possibly because they are monoclonal antibodies and are more specific for the target molecules [54]. The LODs obtained are in the picomolar range, demonstrating the high detectability achieved with the antibodies developed.

Regarding COL-DMP-BSA, the IC50s are similar between the antisera, with Rb66 being the best. The IC50 values obtained are lower than 10 nM, similar to values described in the literature for ELISA-based studies, such as the determination of Colistin in animal feed with an IC 50 of 8.39 nM [62]. For MER-DCC-BSA, there was no evidence of competition for Rb 160–Rb 161 and the IC50 for Rb162 is the highest of all IC50 and LOD antibiotics studied. According to the above, it can be deduced that it is the one with the least detectability compared to the rest of the antibiotics analyzed. Studies with HPLC have been found where it was possible to determine Meropenem in peritoneal fluid with a LOD of 26.07 nM [63], which is closer to the LOD given by immunoassay.

DAP-DMP-BSA Rb 155 and Rb 156 give similar IC50 and LOD values, and on the contrary, Rb154 shows higher IC50, being less specific antisera; in contrast with Rb156 DAP-DCC-BSA, the IC50 and LOD were lower and were not evidenced competition for Rb154. According to reports in the literature, Daptomycin was quantified in human plasma by HPLC with an LOD of 309 nM by HPLC-UV [64] compared to the much lower LOD found in ELISA assays of 0.25 nM. The quantification with the antiserum became more specific.

According to these results, it is possible to determine that most of the antibodies obtained have some degree of detectability with the antibiotics. However, some antisera present greater noise or low specificity, which makes it impossible to determine parameters such as IC50 and LOD. Likewise, it is possible to determine that the most specific antibodies are those of Vancomycin and the least specific are those of Meropenem. When compared with the literature, this is mainly based on the production of some mostly monoclonal antibodies for the quantification of antibiotics in food but not in body fluids or on HPLC techniques that, being more robust, specific and requiring a specialized laboratory, tend to have lower LOD.

For this reason, the antibodies developed in this work would be of great use at the clinical level, to quantify last-line antibiotic molecules, in a simpler and faster way.

## 5. Conclusions

Immunogens were developed for the production of antibodies against Vancomycin, Colistin, Meropenem, and Daptomycin in rabbits. Polyclonal antibodies with good detectability were obtained. To improve detectability in ELISA assays, it was necessary to develop heterologous antigens, which made it possible to determine the antigen–antibody binding with greater certainty, largely eliminating the background noise of the assays in addition to evidencing competence in competitive ELISA assays. It is important to note that among the antibiotics, it was Vancomycin that obtained the lowest IC_50_.

In summary, it has been possible to obtain antibodies against “last-line” antibiotics such as Vancomycin, Colistin, Meropenem, and Daptomycin, which could be used to carry out therapeutic monitoring of drugs using ELISA-type immunoassays or electrochemical and optical biosensors. Obtaining antibodies against antibiotics is very promising for therapeutic measures in patients treated with antibiotics that allow a favorable clinical outcome. This could contribute to the decrease in antibiotic resistance.

## Figures and Tables

**Table 1:** Hapten density for BSA bioconjugates.

Coupling Method	BioConjugate	MW (Conjugate) Da	Hapten Density (δ)
	BSA	66,432	-
EDC/NHS	COL-EDC-BSA	82,769	14
	VAN-EDC-BSA	71,195	3
	DAP-EDC-BSA	No result	No result
	MER-EDC-BSA	74,680	22
DMP	COL-DMP-BSA	72,000	5
	DAP-DMP-BSA	71,565	3
DCC/NHS	VAN-DCC-BSA	71,714	4
	MER-DCC-BSA	75,445	23
	DAP-DCC-BSA	91,116	15

**Table 2:** Chosen concentrations to perform competitive ELISA.

Antibiotic	Antibody	Antisera Dilution	Antigen Concentration (µg/mL)	Absorbance (450 nm)
Vancomycin (VAN-DCC-BSA)	Rb157	1/4000	2.5	0.8684
	Rb158	1/16,000		0.9435
	Rb159	1/4000		0.9801
Colistin (COL-DMP-BSA)	Rb66	1/1000	2.5	0.9480
	Rb67			0.7506
Meropenem (MER-DCC-BSA)	Rb162	1/4000	2.5	1.0649
Daptomycin (DAP-DMP-BSA)	Rb154	1/20,000	0.5	1.0514
	Rb155	1/10,000	0.0625	0.9003
	Rb156		0.01562	1.0426
Daptomycin (DAP-DCC-BSA)	Rb154	1/64,000	5	1.0489
	Rb155	1/32,000	1.25	1.1917
	Rb156	1/32,000	1.25	1.0569

**Table 3:** Parameters of the different competitive ELISAs obtained with the different antisera and antigens.

Antisera	Antigen	Amax	Amin	IC_50_ (nM)	Slope	LOD (nM)
Rb157	VAN-DCC-BSA	101.5	9.97	0.05	−0.60	0.002
Rb158		99.88	−0.57	0.3	−0.63	0.009
Rb159		99.35	−0.22	0.29	−0.57	0.006
Rb66	COL-DMP-BSA	146.9	−58.2	7.56	-	0.003
Rb67		216	4.62	8.75	-	0.002
Rb162	MER-DCC-BSA	1.46	0.14	183.6	−1.19	23.9
Rb154	DAP-DMP-BSA	96.34	−1.17	61.75	−0.72	1.568
Rb155		95.29	−0.85	22.27	−0.63	0.257
Rb156		88.41	1.44	23.52	−0.79	1.433
Rb154	DAP-DCC-BSA	-	-	-	-	-
Rb155		95.63	−0.09	42.52	−0.72	0.937
Rb156		85.50	1.46	13.82	−0.99	0.952

## Data Availability

The original contributions presented in the study are included in the article/supplementary material, further inquiries can be directed to the corresponding author/s.

## Supplementary Materials

The following supporting information can be downloaded at: https://www.mdpi.com/article/10.3390/antibiotics13070600/s1, Figure S1: MALDI-TOFF mass spectral of haptens; Figure S2: Antibody titer by non-competitive indirect ELISA; Table S1: Bioconjugates obtained according hapten and protein combination and conjugation methodology employed; Table S2: Concentrations chosen to perform competitive ELISA with homologous competitors.
