# Supplementary material for: Development and ELISA Characterization of Antibodies against the Colistin, Vancomycin, Daptomycin, and Meropenem: A Therapeutic Drug Monitoring Approach
Source: Antibiotics (Basel). 2024 Jun 27;13(7):600. doi: 10.3390/antibiotics13070600 (PMC11273741; doi:10.3390/antibiotics13070600)
Supplement: Supplementary file 1 [file antibiotics-13-00600-s001.zip › Figure S1.pdf]

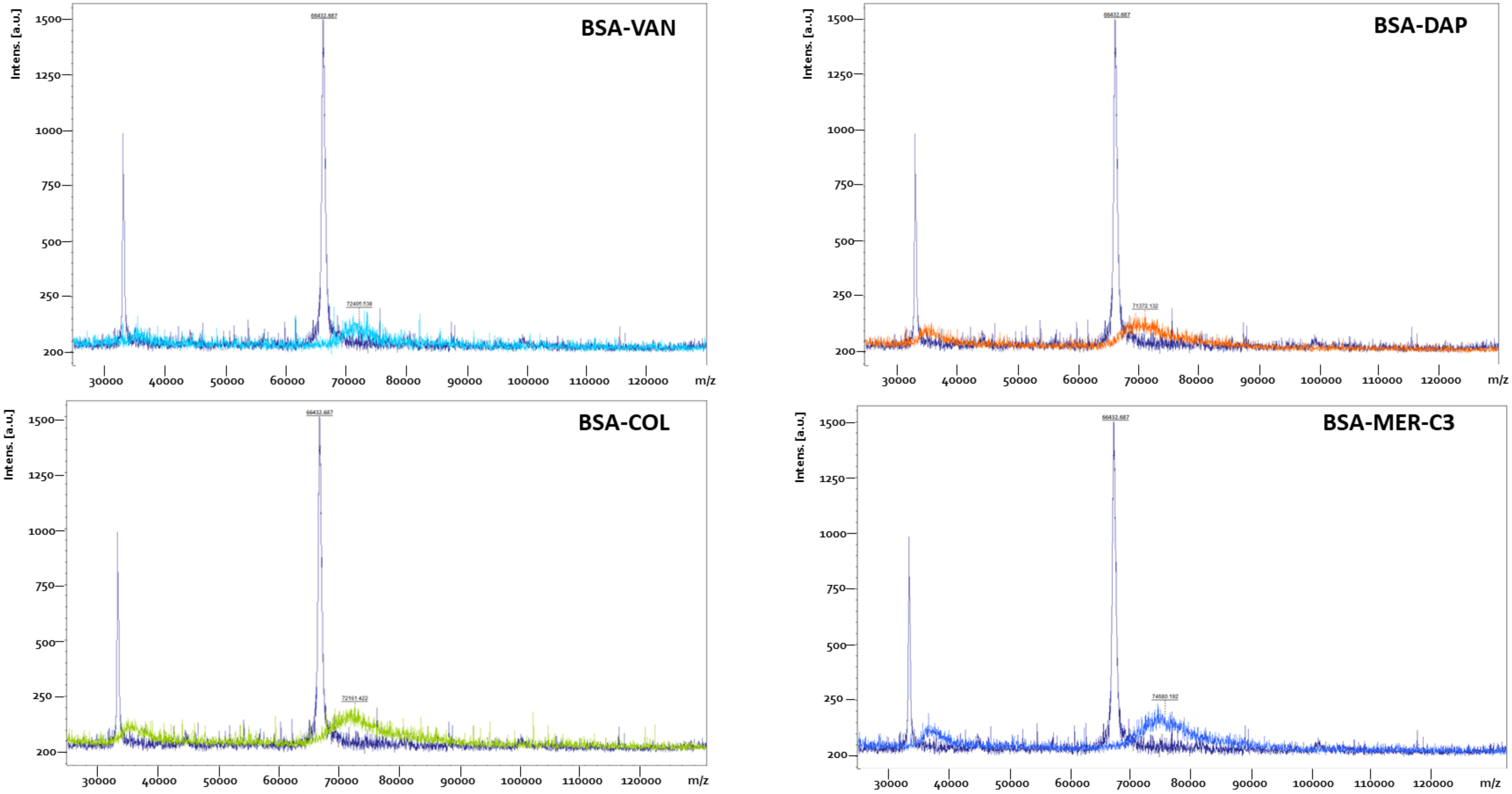

Figure S1. MALDI-TOFF mass spectral of haptens BSA-VAN (Vancomycine), BSA-DAP (Daptomycine), BSA-COL (Colistine) and BSA-MER-C3 (Meropenem). The number of haptens linked to BSA was directly proportional to linker: BSA molar ratios.
