# Supplementary material for: Development and ELISA Characterization of Antibodies against the Colistin, Vancomycin, Daptomycin, and Meropenem: A Therapeutic Drug Monitoring Approach
Source: Antibiotics (Basel). 2024 Jun 27;13(7):600. doi: 10.3390/antibiotics13070600 (PMC11273741; doi:10.3390/antibiotics13070600)
Supplement: Supplementary file 1 [file antibiotics-13-00600-s001.zip › Figure S2.pdf]

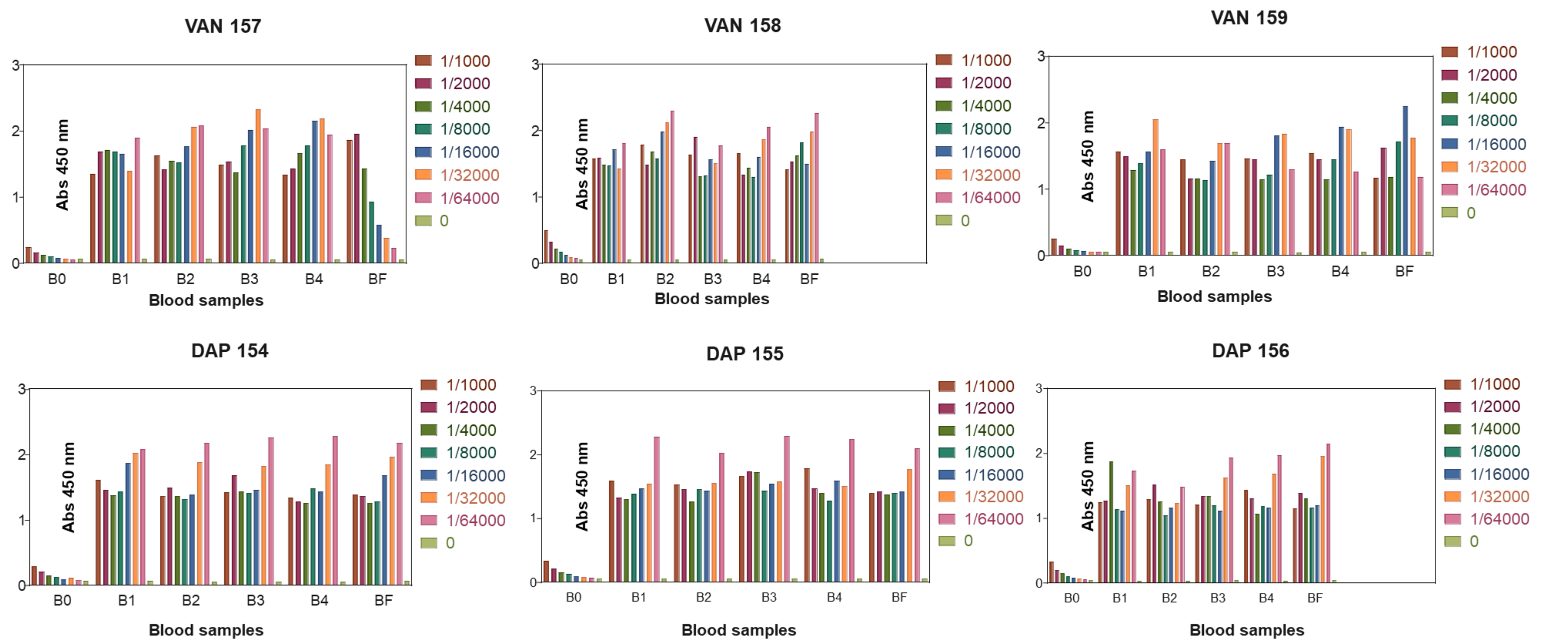
VAN 157
VAN 158
VAN 159
DAP 154
DAP 155
DAP 156
Abs 450 nm
Blood samples
B0
B1
B2
B3
B4
BF
1/1000
1/2000
1/4000
1/8000
1/16000
1/32000
1/64000
0

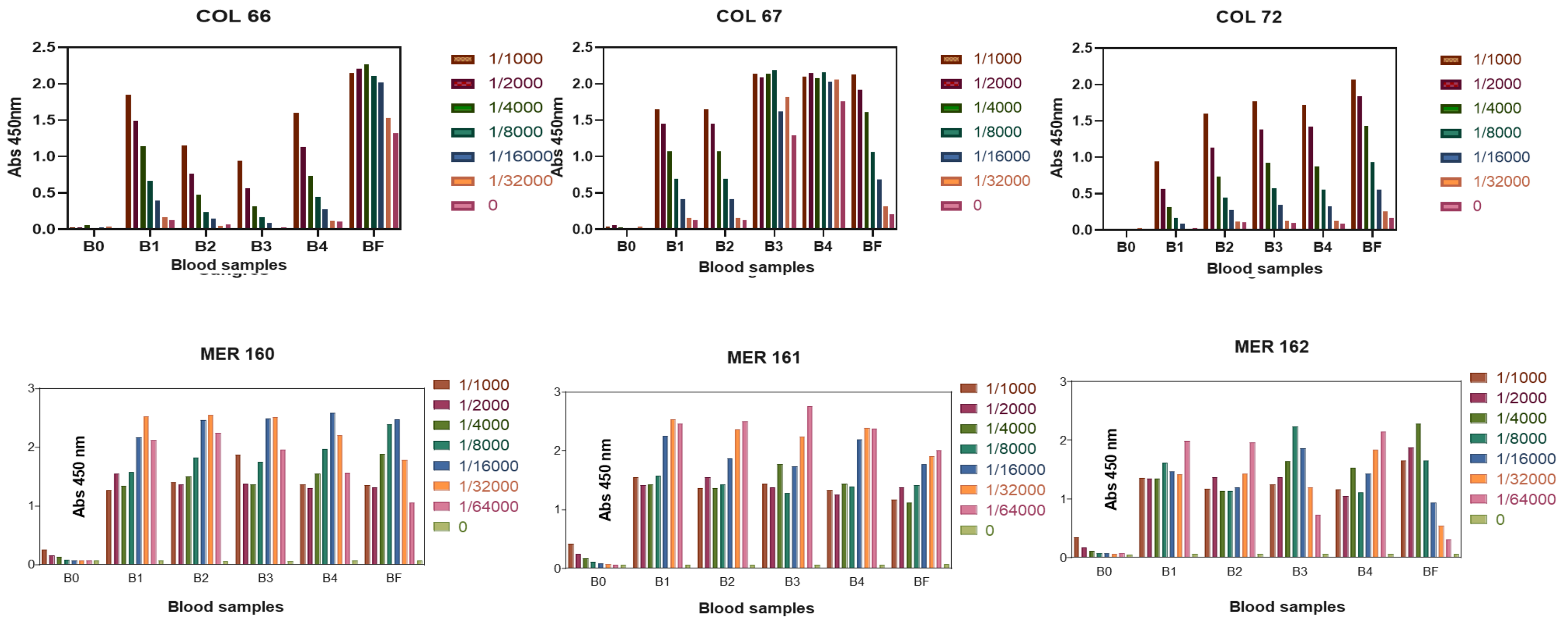

Figure S2. Antibody titer by non-competitive indirect ELISA. Time B0 (preimmune antiserum) before immunization; Time BF: 6 months, corresponding to the last immunization. Plates were coated at 1µ/mL of competitor bioconjugate and antisera were diluted 64,000-fold.
