# Supplementary material for: Development and ELISA Characterization of Antibodies against the Colistin, Vancomycin, Daptomycin, and Meropenem: A Therapeutic Drug Monitoring Approach
Source: Antibiotics (Basel). 2024 Jun 27;13(7):600. doi: 10.3390/antibiotics13070600 (PMC11273741; doi:10.3390/antibiotics13070600)
Supplement: Supplementary file 1 [file antibiotics-13-00600-s001.zip › Table S1. Bioconjugates obtained according hapten and protein combination and conjugation methodology employed.pdf]

Table S1. Bioconjugates obtained according hapten and protein combination and conjugation methodology employed.

Type of bioconjugate	Protein	Method	Colistin	Vancomycin	Daptomycin	Meropenem
Coating antigen	BSA	EDC/NHS	COL-EDC-BSA	VAN-EDC-BSA	DAP-EDC-BSA	MER-EDC-BSA
		DCC/NHS	----	VAN-DCC-BSA	DAP-DCC-BSA	MER-DCC-BSA
		DMP	COL-DMP-BSA	---	DAP-DMP-BSA	---
Immunogen	HCH	EDC/NHS	COL-EDC-HCH	VAN-EDC-HCH	DAP-EDC-HCH	MER-EDC-HCH

EDC: 1-ethyl-3-(dimethylaminopropyl)carbodiimide hydrochloride; NHS: N-hydroxysuccinimide; BSA: Bovine serum albumin; HCH: horseshoe crab hemocyanin; COL: Colistine; VAN: Vancomycine; DAP: Daptomycine; MER: Meropenem
