# Supplementary material for: Development and ELISA Characterization of Antibodies against the Colistin, Vancomycin, Daptomycin, and Meropenem: A Therapeutic Drug Monitoring Approach
Source: Antibiotics (Basel). 2024 Jun 27;13(7):600. doi: 10.3390/antibiotics13070600 (PMC11273741; doi:10.3390/antibiotics13070600)
Supplement: Supplementary file 1 [file antibiotics-13-00600-s001.zip › Table S2. Concentrations chosen to perform competitive ELISA with homologous competitors.pdf]

Table S2. Concentrations chosen to perform competitive ELISA with homologous competitors

Antibiotic	Antibody	Dilution Antisera	Concentration antigen (µg/mL)	Absorbance (450 nm)
**Vancomycin (VAN-EDC-BSA)**	Rb157	1/4000	2.5	2.0654
	Rb158			2.0546
	Rb159			2.0534
**Colistin (COL-EDC-BSA)**	Rb66	1/1000	0.5	2.8945
	Rb67			2.5678
	Rb72	1/2000		1.6547
**Meropenem (MER-EDC-BSA)**	Rb162	1/20000	2.5	1.8654
**Daptomycin (DAP-EDC-BSA)**	Rb154	1/20000	0.5	1.0234
	Rb155			2.0285
	Rb156			1.03456
